# Single-Cell Omics Advances in Understanding Tissue Development and Complex Trait Formation in Sheep and Goats

**DOI:** 10.3390/ani16131948

**Published:** 2026-06-23

**Authors:** Jianfang Wang, Haobin Ma, Diba Dedacha Jilo, Abebe Belete Kuraz, Juntao Guo, Yajuan Li, Xiaogao Diao, Bouabid Badaoui, Rui Su, Yongbin Liu

**Affiliations:** 1 College of Animal Science, Inner Mongolia Agricultural University, Inner Mongolia Autonomous Region, Hohhot 010018, China; jfwang@imau.edu.cn (J.W.); liyj@imau.edu.cn (Y.L.); 20250054dxg@imau.edu.cn (X.D.); 2 College of Animal Science and Technology, Northwest A&F University, Yangling 712100, China; mahaobin@nwafu.edu.cn (H.M.); milkidiba2023@gmail.com (D.D.J.); beleab2@gmail.com (A.B.K.); juntao@nwafu.edu.cn (J.G.); 3 Department of Animal Science, Bule Hora University, Bule Hora P.O. Box 144, Ethiopia; 4 Department of Animal Science, Werabe University, Werabe P.O. Box 46, Ethiopia; 5 Laboratory of Biodiversity, Ecology and Genome, Department of Biology, Faculty of Sciences Rabat, Mohammed V University, Rabat 10106, Morocco; bouabidbadaoui@gmail.com; 6 Sino-Arabian Joint Laboratory of Sheep and Goat Germplasm Innovation, Inner Mongolia Autonomous Region, Hohhot 010018, China; 7 Inner Mongolia Key Laboratory of Sheep & Goat Genetics Breeding and Reproduction, Inner Mongolia Autonomous Region, Hohhot 010018, China; 8 China-Mongolia Biomacromolecule Application “Belt and Road” Joint Laboratory, Inner Mongolia Autonomous Region, Hohhot 010018, China; 9 Key Laboratory of Mutton Sheep Genetics and Breeding, Ministry of Agriculture and Rural Affairs, Inner Mongolia Autonomous Region, Hohhot 010018, China

**Keywords:** single-cell omics, sheep and goats, tissue development, trait formation, precision breeding

## Abstract

Single-cell omics technologies have transformed the study of cellular heterogeneity, enabling high-resolution analysis of tissue development and complex traits. In sheep and goats, these approaches have been applied to skin, hair follicles, reproductive organs, metabolic tissues, and adipose tissue, revealing cell type-specific regulatory programs underlying traits such as wool quality, fertility, growth, and fat deposition. However, most studies rely on single-cell RNA sequencing (scRNA-seq) and are limited by incomplete genome annotation, insufficient coverage of production traits, and weak integration with population genetics, restricting their application in molecular breeding. This review summarizes advances in single-cell omics in sheep and goats, focusing on tissue development and trait formation. We further discuss emerging strategies that integrate single-cell multi-omics, spatial transcriptomics, and population genetics to resolve regulatory mechanisms in a cell type-specific and spatially informed context. Finally, we discuss CRISPR/Cas9-based validation to link genotype and phenotype, accelerating gene discovery and precision breeding in small ruminants.

## Simple Summary

Sheep and goats provide valuable products such as wool, meat, and milk. The quality of these products is closely linked to how cells work together in tissues like skin, reproductive organs, and adipose. New technologies now allow scientists to study biology at single-cell resolution, helping to uncover how genes control traits such as wool growth, reproduction, fat deposition, and overall animal performance. This review summarizes recent discoveries in sheep and goats using these single-cell approaches. We also discuss current challenges and suggest promising strategies to better understand the cellular basis of economically important traits. Ultimately, this knowledge can support breeding efforts to improve animal health and productivity, benefiting farmers.

## 1. Introduction

**Figure 1:**
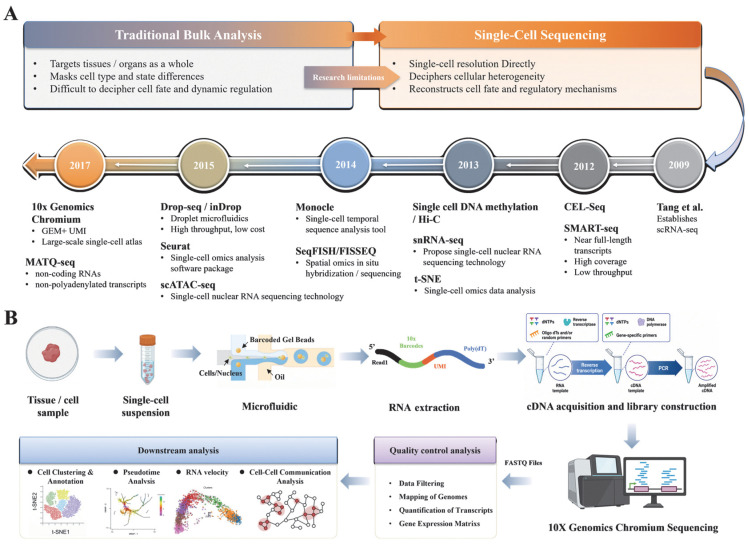
Schematic overview of the single-cell omics development and workflow. (**A**) Timeline of key technological advancements in single-cell sequencing. (**B**) Experimental principle and data analysis pipeline of scRNA-seq. (Materials sourced from bioRender).

The development of complex traits in multicellular organisms is driven by the coordinated behavior of diverse cell populations across spatial and temporal scales. In livestock species such as sheep and goats, economically important traits including muscle growth, adipose deposition, wool fiber characteristics, reproductive performance, and metabolic efficiency are not determined solely by bulk tissue properties. Instead, they arise from dynamic processes involving cell fate decisions, lineage differentiation, and intercellular communication. However, traditional population-level approaches, such as bulk RNA sequencing and proteomics, average signals across heterogeneous cell populations, thereby obscuring critical cell-type-specific regulatory mechanisms and limiting our understanding of complex trait formation [1,2]. Recent advances in single-cell omics technologies have transformed our ability to investigate biological systems by resolving cellular heterogeneity, developmental trajectories, and regulatory networks at unprecedented resolution (Figure 1A). By enabling the characterization of individual cells, these approaches provide unique opportunities to identify functional genes, regulatory pathways, and cellular interactions that underlie economically important traits. Such information has important implications for livestock breeding, where long generation intervals and relatively low reproductive efficiency often make conventional breeding programs time-consuming and costly [3]. The integration of single-cell omics with genomic selection, molecular markers, and other omics datasets may improve the accuracy of genetic evaluation at early developmental stages, facilitate the discovery of breeding biomarkers, and accelerate genetic gain [4].

As representative small ruminants with substantial agricultural and biological importance, sheep and goats serve as valuable models for studying the genetic regulation of economically important traits, including fiber production, reproduction, growth, and metabolic adaptation. Recent single-cell studies have begun to characterize the cellular composition and regulatory landscapes of key tissues, particularly hair follicles, reproductive organs, digestive tissues, adipose depots, and mammary glands (Table 1), revealing novel cell populations and regulatory mechanisms associated with phenotypic variation. However, current research remains unevenly distributed across tissues and technologies. Hair follicle biology and reproductive tissues have received the greatest attention, whereas muscle development, immune-related processes, rumen physiology, and cross-organ regulatory mechanisms remain comparatively underexplored. Moreover, most studies rely on scRNA-seq, while the application of single-nucleus sequencing, spatial transcriptomics, and multi-omics integration is still limited. These gaps highlight the need for more comprehensive livestock cell atlases and integrative analytical frameworks.

In this review, we first outline the development of single-cell sequencing technologies and analytical pipelines. We then systematically summarize their applications in key tissues related to economically important traits in sheep and goats. Furthermore, we discuss current limitations in data integration and functional interpretation, and propose future directions that integrate single-cell omics with population genetics and genome editing. Through this approach, we aim to provide a conceptual and methodological framework for advancing functional genomics and precision breeding in small ruminants.

## 2. Evolution of Single-Cell Sequencing Technologies

### 2.1. Early Low-Throughput scRNA-Seq Methods

The field of single-cell omics originated from the initial establishment of transcriptome sequencing by Tang et al. in 2009 [15]. In this early study, sequencing libraries were generated using the SOLiD sequencing system (Thermo Fisher Scientific, Waltham, MA, USA). Since then, scRNA-seq has rapidly evolved into a core technology for dissecting cellular heterogeneity and complex biological systems. Nevertheless, early scRNA-seq approaches faced several limitations, including the inability to capture non-polyadenylated transcripts, technical challenges in isolating individual cells, and amplification-induced biases. To address these issues, Kivioja et al. introduced unique molecular identifiers (UMIs), consisting of short random sequences attached to individual DNA molecules before PCR amplification, which enabled correction of amplification bias during library preparation [16]. In the same year, Islam et al. developed the single-cell tagged reverse transcription (STRT) method at the Karolinska Institutet (Stockholm, Sweden), which employs a template-switching mechanism during reverse transcription to introduce defined sequences at the 3′ ends of cDNA molecules, thereby reducing intercell amplification bias and enabling fewer PCR cycles [17]. Subsequently, advances in sequencing platforms and amplification strategies led to the development of low-throughput linear amplification methods such as Cell Expression by Linear Amplification and Sequencing (CEL-Seq) and Switching Mechanism at the 5′ End of the RNA Transcript (SMART-seq) in 2012 (Figure 1A, Table 2). CEL-Seq integrates UMIs during reverse transcription to minimize PCR preference effects and improve quantitative accuracy [18], whereas SMART-seq enables near full-length transcript coverage, facilitating detailed analyses of cell type-specific transcript structures and allele-specific expression [19,20]. However, these early approaches were limited in throughput, typically profiling only dozens to hundreds of cells, which restricted their application to systems with low cellular complexity, such as early embryonic development, and limited their utility in heterogeneous tissues [21,22].

### 2.2. Emergence of High-Throughput Platforms

The advent of droplet-based microfluidics marked a major breakthrough by dramatically increasing throughput while reducing cost [24]. In 2015, droplet microfluidic technologies represented by Drop-seq and inDrop enabled random co-encapsulation of cells with barcoded beads, allowing transcriptomic profiling of tens of thousands of cells in a single experiment [25,26]. Building on this principle, 10× Genomics (Pleasanton, CA, USA) introduced the Chromium Single Cell platform in 2017, based on Gel Bead-In-Emulsion (GEM) technology. By integrating automated microfluidics, UMIs, and optimized library construction workflows, this platform further improved data quality, reproducibility, and scalability, facilitating large-scale single-cell atlas projects [27,28].

In parallel, multiple annealing and dC-tailing-based quantitative single-cell RNA sequencing (MATQ-seq) employed random priming strategies to enable comprehensive capture of total RNA, including non-coding and non-polyadenylated transcripts [29]. Additional high-throughput platforms based on microwell arrays and combinatorial indexing were subsequently developed, including gene expression cytometry sequencing (Cyto-Seq) [30], single-cell RNA sequencing in a well-based format (SeqWell) [31], microwell-based single-cell RNA sequencing (Microwell-Seq) [32], single-cell combinatorial indexing RNA sequencing (sci-RNA-Seq) [33], and split-pool ligation-based transcriptome sequencing (SPLiT-Seq) [34]. More recently, in 2023, Xu et al. introduced a novel single-nucleus RNA-seq (snRNA-seq) approach combining random priming with droplet microfluidics, enabling high-throughput transcriptional profiling of individual nuclei and overcoming challenges associated with isolating intact cells from frozen or difficult-to-dissociate tissues [35].

Despite these advantages, high-throughput approaches often rely on 3′ or 5′ end counting strategies and typically provide limited information on full-length transcript structure. Therefore, a trade-off exists between sequencing depth and cell number, which should be considered in experimental design.

### 2.3. Expansion Beyond Transcriptomics: Epigenomics and Spatial Technologies

While scRNA-seq provides valuable information on gene expression, it captures only one layer of cellular regulation. To achieve a more comprehensive understanding of cellular states, single-cell technologies have expanded into the epigenomic and spatial domains, including DNA methylation, histone modifications, chromatin accessibility, and three-dimensional chromatin conformation. The development of scATAC-seq in 2015 enabled chromatin accessibility profiling at single-cell resolution, offering a powerful tool for identifying cis-regulatory elements and inferring transcription factor activity [36]. Subsequently, single-cell CUT & RUN and CUT & Tag techniques further reduced background noise and enabled high-resolution mapping of epigenetic modifications and protein–DNA interactions at the single-cell level [37], thereby laying the foundation for constructing cell type-specific regulatory networks.

In recent years, spatial transcriptomics has expanded single-cell research from cell identity characterization to spatial tissue architecture reconstruction. Platforms such as 10× Visium Spatial Gene Expression platform (10× Genomics, Pleasanton, CA, USA), Slide-seqV2, and Stereo-seq (BGI-Research, Shenzhen, China) enable transcriptomic profiling while preserving spatial information, allowing systematic mapping of cellular organization and intercellular communication within tissues and overcoming the spatial limitations of conventional scRNA-seq [38]. Building on these advances, single-cell multi-omics analysis strategies are gradually becoming a research focus. By jointly profiling the transcriptome, epigenome, and spatial information from the same cell or adjacent tissue sections, these approaches not only allow for the integrative analysis of cell states, regulatory mechanisms, and spatial microenvironments, but also provide a more systematic and three-dimensional research framework. This framework facilitates a deeper understanding of tissue developmental zoning, the formation of functional heterogeneity, and the spatial localization of key regulatory nodes [39].

## 3. Analytical Pipelines for Single-Cell Data Processing

### 3.1. Tissue Dissociation Strategies for Single-Cell Analysis

The generation of high-quality single-cell or single-nucleus suspensions is a critical prerequisite for successful scRNA-seq analyses [40]. Because different tissues exhibit distinct extracellular matrix (ECM) compositions, cell–cell adhesion properties, and structural complexities, tissue-specific dissociation protocols must be carefully optimized to minimize cellular stress, preserve transcriptomic integrity, and ensure representative recovery of diverse cell populations [41,42,43].

Most tissue dissociation protocols combine enzymatic digestion with mechanical disruption. Enzymatic digestion is typically performed at 37 °C for 20–120 min, depending on tissue type, density, and ECM composition. In general, shorter digestion times are preferred because prolonged exposure to proteolytic enzymes can compromise cell viability and induce artificial stress-response transcriptional programs [44]. Among the enzymes used, collagenases are the most common owing to their ability to degrade ECM components efficiently. Collagenase type I is frequently employed for adipose tissue [45,46] and mammary gland dissociation [14], whereas collagenase type II is often used for oral mucosa [47] and pancreatic tissues [48]. Collagenase type IV, which possesses relatively low tryptic activity, is generally preferred for epithelial-rich tissues such as testis [7], skin [6], lacrimal and salivary glands [49]. Additional enzymes, including Dispase, hyaluronidase, Accutase, and TrypLE, are frequently incorporated to improve cell recovery, reduce cell aggregation, and facilitate the dissociation of tissue-specific structures [44,50,51]. DNase is another commonly used component during tissue dissociation and exists primarily in two forms, DNase I and DNase II, which differ substantially in their biological functions. DNase II (is mainly involved in lysosome-mediated degradation of apoptotic DNA during phagocytosis and is therefore unsuitable for single-cell preparation procedures [52,53]. In contrast, DNase I is routinely included in tissue digestion protocols because it degrades extracellular DNA released from damaged or dead cells during enzymatic dissociation, thereby preventing cell clumping and improving the quality of single-cell suspensions without triggering apoptotic pathways [44].

Following enzymatic digestion, tissues are mechanically dissociated using gentle pipette trituration, Dounce homogenization, tissue grinders, or automated systems such as the gentleMACS Dissociator [42]. The resulting suspensions are typically filtered through 40–100 μm cell strainers to remove debris, undigested tissue fragments, and cellular aggregates. Cell viability represents a key determinant of sequencing quality, and most scRNA-seq workflows recommend viability levels above 70–85% before library preparation. Additional purification steps may be performed using fluorescence-activated cell sorting (FACS) [54] or magnetic-activated cell sorting (MACS) [42] to enrich target cell populations and remove dead cells, doublets, erythrocytes, and other unwanted cellular contaminants. FACS is particularly advantageous for isolating rare cell populations or obtaining highly purified cell fractions, although prolonged sorting procedures may increase cellular stress and potentially alter transcriptional profiles.

Different livestock tissues present unique dissociation challenges that require specialized optimization strategies. Skin and hair follicle tissues often necessitate collagenase- and Dispase-based digestion combined with microdissection techniques to preserve follicular cell populations. Reproductive tissues such as ovary and testis require carefully controlled digestion conditions to avoid damage to granulosa cells, germ cells, Sertoli cells, and Leydig cells. Dense epithelial tissues, including rumen epithelium and intestinal mucosa, frequently benefit from additional enzymatic treatments and mechanical disruption to improve cell yield and recovery efficiency. Mammary gland tissues contain abundant fibro-adipose components and often require prolonged digestion or alternative nuclei-based approaches. Skeletal muscle and adipose tissues are among the most challenging samples because multinucleated myofibers and mature adipocytes are difficult to capture using droplet-based scRNA-seq platforms and are particularly susceptible to dissociation-induced transcriptional artifacts. For highly fibrous, fragile, archived frozen, or otherwise difficult-to-dissociate tissues, snRNA-seq has emerged as an effective alternative to conventional scRNA-seq [43,55,56]. Nuclei isolation is generally performed under cold conditions (4 °C) using detergent-based lysis buffers, thereby minimizing the transcriptional perturbations commonly induced by enzymatic digestion and prolonged tissue processing. Collectively, careful optimization of tissue dissociation, cell purification, and quality-control procedures is essential for generating representative cellular profiles and ensuring the biological reliability and reproducibility of livestock single-cell datasets.

### 3.2. Single-Cell Sequencing and Anlysis Workflow

Single-cell sequencing begins with the preparation of single-cell or single-nucleus suspensions from tissues or cultured cells. Individual cells are encapsulated into microdroplets (gel beads-in-emulsion, GEMs), where cDNA molecules are labeled with cell-specific barcodes and unique molecular identifiers (UMIs), enabling accurate transcript quantification at the single-cell level (Figure 1B). Following Illumina short-read sequencing, the resulting libraries generate FASTQ files, typically including read 1 (R1), read 2 (R2), and index sequences. Since both scRNA-seq and bulk RNA-seq generate data in FASTQ format, they share similar preprocessing procedures, including read filtering, alignment to reference genomes, and transcript quantification. Accordingly, many computational pipelines and software tools developed for bulk RNA-seq are also applicable to scRNA-seq data [57].

With the rapid advancement of sequencing technologies, several single-cell platforms have been developed, including 10× Genomics Chromium system (10× Genomics, Pleasanton, CA, United States), Nadia (Dolomite Bio, Royston, Hertfordshire, UK), Illumina Bio-Rad ddSEQ (Bio-Rad, Hercules, CA, USA), BD Rhapsody (BD Biosciences, San Jose, CA, USA), ICELL8 Single-cell System (Takara, Mountain View, CA, USA), and Fluidigm C1 (Fluidigm, South San Francisco, CA, USA) [58]. Among these, 10× Genomics Chromium system is the most widely adopted because of its high sensitivity, scalability, and ability to capture a large proportion of cells from heterogeneous samples [59]. The accompanying Cell Ranger software performs automated read preprocessing, genome alignment using the STAR algorithm, UMI deduplication, and gene expression quantification directly from raw sequencing data [60]. Across the reviewed sheep and goat studies, Cell Ranger versions varied substantially (e.g., v2.2.0–v7.2.0), highlighting the continuous development of single-cell bioinformatics pipelines and the importance of reporting software versions to ensure reproducibility. The pipeline outputs key files including the filtered_feature_bc_matrix (gene-cell expression matrix), barcodes.tsv (cell barcodes), features.tsv (gene names/IDs), metrics_summary.csv (sample quality statistics), possorted_genome_bam.bam (aligned sequences), and web_summary.html (sample quality and analysis overview). Multi-sample analysis can be achieved by using the CellRanger aggr function to aggregate and correct for batch effects in the expression matrix, thereby eliminating inter-experiment variations. The resulting gene–cell expression matrix serves as the foundation for downstream analyses using tools such as Seurat, Monocle, and related packages (Figure 1B).

Prior to downstream biological interpretation, quality control is performed to remove low-quality or stressed cells and potential doublets based on metrics such as detected gene and UMI counts per cell, mitochondrial read proportions, and doublet prediction scores [61]. Highly variable genes (HVGs) are then selected to reduce computational complexity and enrich biologically informative signals. Principal component analysis (PCA) is applied for linear dimensionality reduction, followed by non-linear visualization methods such as t-distributed stochastic neighbor embedding (t-SNE) [62] and uniform manifold approximation and projection (UMAP) [63]. Graph-based clustering algorithms, including Louvain and Leiden methods implemented in Seurat, are subsequently applied to identify transcriptionally similar cell populations and characterize tissue cellular heterogeneity [64]. Cell type annotation is then performed using known marker genes, reference databases such as CellMarker and PanglaoDB, and automated tools including SingleR and scmap [65].

Beyond static characterization of cell populations, single-cell data enable reconstruction of dynamic biological processes. Trajectory inference methods such as Monocle [66], Slingshot [67], and PAGA [68] enable the ordering of cells along developmental or differentiation pathways, providing insights into lineage relationships and transitional cell states. RNA velocity analysis further predicts future transcriptional states by leveraging spliced and unspliced transcript ratios, enhancing inference of directional cell fate transitions [69]. In addition, cell–cell communication analyses using tools such as CellPhoneDB, CellChat, CytoTalk, and NicheNet facilitate the reconstruction of intercellular signaling networks and reveal regulatory interactions within tissue microenvironments [70,71,72]. Together, these analytical approaches provide a comprehensive framework for dissecting cellular heterogeneity, developmental trajectories, and intercellular communication.

### 3.3. Cross-Species Reference Mapping and Cell-Type Annotation in Sheep and Goats

Comparative single-cell transcriptomics has emerged as an important approach for investigating evolutionary conservation and species-specific developmental programs. Because genome annotations for sheep (*Ovis aries*) and goat (*Capra hircus*) remain less complete than those available for model organisms, many small-ruminant studies employ cross-species projection strategies to improve cell-type identification. In these approaches, sheep and goat datasets are integrated with well-annotated reference atlases derived from human, mouse, bovine, or porcine tissues. Cross-species annotation generally begins with ortholog mapping, in which livestock genes are converted into corresponding orthologs in the reference species. Databases such as Ensembl BioMart and OrthoDB are commonly used to identify high-confidence one-to-one orthologous genes, thereby minimizing ambiguity caused by duplicated or species-specific genes [73,74].

Several computational frameworks have been developed for cross-species integration and label transfer (Table 3). Seurat employs an anchor-based strategy through the FindTransferAnchors function to project query cells onto annotated references and has become one of the most widely used approaches for cross-species integration [75]. SingleR automatically assigns cell identities based on transcriptomic similarity to reference datasets [76], whereas scmap performs projection of query cells onto reference clusters or individual cells [77]. More recently, CellTypist has incorporated machine-learning classifiers to improve automated cell-type annotation, particularly for immune populations [78].

In addition to label transfer, batch-correction algorithms including Harmony [79], LIGER [80], and Scanorama [81] facilitate integration of datasets generated across species, laboratories, and sequencing platforms. Following automated annotation, predicted cell identities should be validated using conserved marker genes and biological knowledge to ensure reliable interpretation. Collectively, these comparative approaches enable small-ruminant single-cell studies to leverage the extensive cellular resources available for human and model organisms, thereby improving annotation accuracy and facilitating the discovery of conserved and species-specific regulatory mechanisms.

## 4. Single-Cell Insights into Hair Follicle Development and Wool Trait Formation

### 4.1. Hair Follicle Development and Regulation

**Figure 2:**
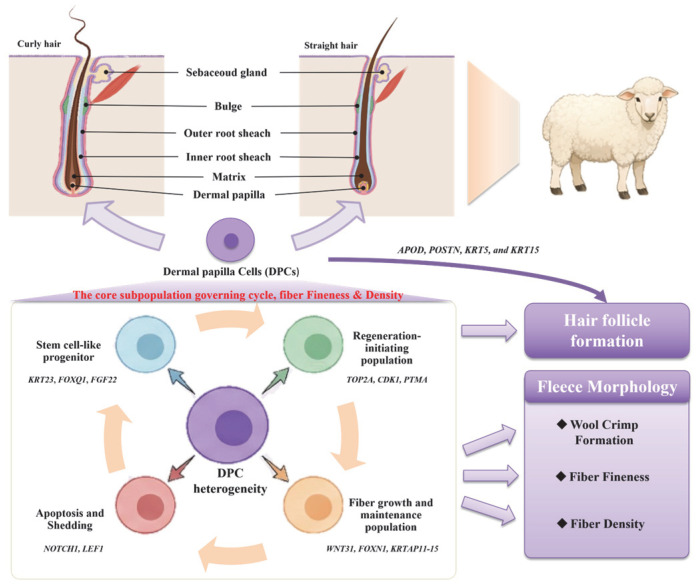
Dermal papilla cell heterogeneity governs hair follicle cycling and wool trait formation in sheep and goats. (Materials sourced from bioRender).

Hair follicles are highly dynamic regenerative mini-organs, whose development and function rely on tightly coordinated interactions among diverse epithelial and mesenchymal populations. Single-cell transcriptomic analyses in sheep and goats have revealed that epithelial lineages (e.g., hair follicle stem cells (HFSCs), matrix cells (MCs), and inner/outer root sheath cells) and mesenchymal niches (e.g., dermal papilla cells (DPCs), dermal sheath cells) exhibit distinct yet interdependent transcriptional states that collectively regulate follicle growth, cycling, and fiber traits. scRNA-seq profiling across key embryonic stages in Shanbei White Cashmere goats (E60–E120) generated a continuous developmental atlas encompassing follicle induction, morphogenesis, and terminal differentiation, identifying major cell populations including dermal, epithelial, endothelial, muscle, and immune cells [5]. Transcription factors such as *GATA3*, *PRDM1*, and *LEF1* were shown to control lineage specification within the follicle [82]. Notably, DPCs exhibit marked intra-population heterogeneity, with four intermediate states displaying stage-specific functions across the hair cycle: a stem cell-like progenitor population (*KRT23*, *FOXQ1*, *FGF22*), a regeneration-initiating population responsible for new cycles and follicle migration (*TOP2A*, *CDK1*, *PTMA*), a fiber growth and maintenance population (*WNT31*, *FOXN1*, *KRTAP11-15*), and a regression/apoptosis-associated population (*NOTCH1*, *LEF1*) (Figure 2) [83]. Additionally, MCs are enriched for Wnt signaling and apoptosis-related genes, including *KRT19*, *MSX2*, and *LEF1*, highlighting their role in coordinating follicle proliferation and cyclic remodeling [84]. Collectively, these findings indicate that hair follicle development and regeneration are driven by continuous differentiation trajectories, dynamic cell–cell interactions, and lineage-specific regulatory programs, providing a refined cellular and molecular framework for understanding wool trait formation and cashmere fiber quality in ruminants.

### 4.2. Fine Wool Trait Formation

Beyond structural and developmental insights, single-cell approaches have begun to elucidate the molecular regulatory underlying economically important wool traits, particularly fiber fineness and density. Single-cell transcriptomic profiling of hair follicle cells from Hu sheep with curly and straight wool identified 19 distinct cell clusters and reconstructed the differentiation trajectory of matrix cells into hair shaft and inner root sheath (IRS) lineages, highlighting dermal papilla cells (DPCs) as central regulators of wool crimp formation (Figure 2) [6]. This finding suggests that fleece morphology is primarily driven by regulatory heterogeneity within specific follicular cell subpopulations, rather than by keratin gene expression alone. Further evidence supports the conserved role of dermal papilla-related cell populations in fiber trait regulation. In Ordos fine-wool sheep, DPC-enriched genes, including *APOD*, *POSTN*, *KRT5*, and *KRT15*, were identified as key factors associated with primary and secondary follicle formation, providing potential targets for modulating fiber diameter and follicle density [85]. Extending these findings to goats, single-cell transcriptomic analyses identified secondary dermal papilla cells (SDPCs) as a putative cell population associated with cashmere fiber fineness and density [86]. Several genes related to extracellular matrix organization and hair follicle development, such as *COL1A1*, *COL3A1*, *CCDC80*, *S100A4*, and *CXCL8*, were highly expressed in SDPCs from fine-fiber goats. These transcriptional features suggest that SDPCs may play an important role in the regulation of cashmere fiber characteristics. However, further functional studies are required to establish their causal role in determining fiber fineness and density.

## 5. Single-Cell Analysis of Reproductive System Development

### 5.1. Spermatogenesis and Testicular Cell Dynamics

**Figure 3:**
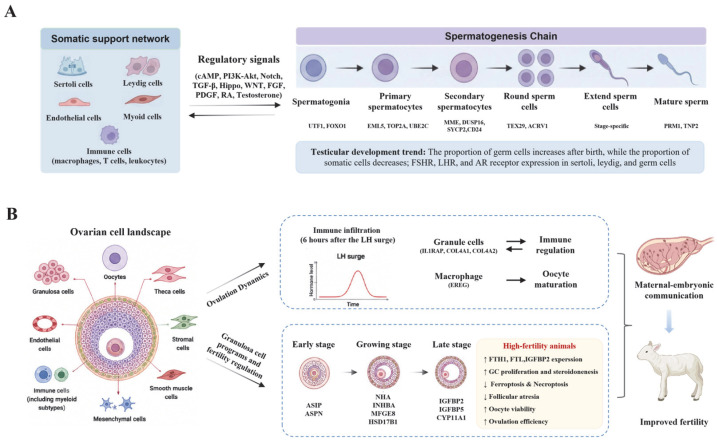
Single-cell transcriptomic analysis of reproductive system development in sheep and goats. (**A**) Somatic support network and spermatogenesis chain in the testis. (**B**) The cellular and molecular regulation during ovarian development and reproduction in sheep and goats. The upward arrow (↑) indicates promotion, whereas the downward arrow (↓) indicates inhibition.

Spermatogenesis is a highly coordinated and tightly regulated process that depends on dynamic interactions between germ cells and diverse somatic cell populations within the testicular microenvironment (Figure 3A). The intrinsic cellular complexity of the testis, together with the continuous developmental transitions from spermatogonia to mature spermatozoa, has historically limited the resolution at which lineage-specific transcriptional programs and cell-type-specific regulatory mechanisms could be accurately characterized. The emergence of single-cell transcriptomic technologies has largely overcome these limitations, enabling unprecedented insights into spermatogenesis and testicular cell dynamics.

Across ovine studies, a conserved cellular framework has been consistently identified, including major germ cell populations (spermatogonia, primary and secondary spermatocytes, spermatids, and sperm) alongside key somatic cell types such as Sertoli and Leydig cells. The identification of canonical marker genes, such as *EZH2*, *SOX18*, *DDX4*, *UCHL1*, and *PCNA*, further supports the robustness of cell-type annotation and highlights the utility of single-cell approaches in defining germ cell lineage progression [87,88]. Beyond this conserved architecture, single-cell analyses have also revealed dynamic and stage-dependent variations in testicular cell composition. In pre-pubertal testes, a greater diversity of somatic cell populations, including immune cells (e.g., macrophages, monocytes, leukocytes and dendritic cells) and structural components such as endothelial and smooth muscle cells, has been observed, reflecting the complexity of the developing testicular microenvironment [87]. In contrast, analyses of adult and sexually mature testes have provided finer resolution of germ cell subtypes, enabling more precise characterization of spermatogenic progression, including the distinction between early and late spermatocytes as well as round and elongated spermatids [88,89]. These differences underscore the dynamic nature of testicular cell populations during postnatal development, where germ cell proportions progressively increase while somatic cell representation declines [90]. In addition, single-cell studies have highlighted key regulatory pathways, such as cAMP, PI3K-Akt, and ECM-receptor interactions [89], as well as the cell-type-specific expression of hormone receptors (e.g., FSHR, LHR, and AR) [90], providing mechanistic insights into the regulation of spermatogenesis.

In dairy goats, single-cell sequencing has further expanded this framework by generating high-resolution testicular atlases that similarly identified multiple germ cell and somatic cell populations, including immune and structural cell types [7]. Beyond cell identification, these studies provided deeper mechanistic insights into spermatogonial stem cell (SSC) maintenance and differentiation, highlighting key signaling pathways such as Notch, TGF-β, Hippo, and pluripotency-related networks [7]. In addition, candidate SSC-specific marker genes, including *TKTL1* and *AES*, were proposed, offering new molecular tools for SSC isolation and functional studies. Subsequent analyses across developmental stages refined the dynamic trajectory from spermatogonia to mature sperm and identified stage-specific genes (e.g., *AMH*, *SOHLH1*, *INHA*, and *ACTA2*), as well as critical regulatory signaling molecules such as testosterone, retinoic acid (RA), PDGF, FGF, and WNT, which collectively contribute to spermatogenesis and the establishment of the testicular niche [8].

Beyond single-species analyses, cross-species single-cell multi-omics approaches have provided a broader evolutionary perspective on gonadal development. By integrating transcriptomic and chromatin accessibility data, comparative studies across goat, pig, macaque, and human have revealed both conserved and species-specific regulatory programs in early gonads. A set of evolutionarily conserved transcription factors, including *TCF3*, *YY1*, *RBPJ*, and *FOXO1*, have been identified, alongside species-specific regulators such as *ISL1*, *ETS2*, *ZFP2*, and *ZNF581* [91]. Moreover, these integrative analyses have enabled systematic dissection of molecular interaction networks between primordial germ cells and gonadal somatic cells, uncovering key cis-regulatory elements and their associated regulatory factors. These findings provide important clues for understanding the mechanisms governing caprine gonadal cell differentiation and their conservation across species.

### 5.2. Ovarian Follicle Development and Ovulation

Single-cell transcriptomics has substantially advanced our understanding of oogenesis, follicular development, and ovulation by enabling high-resolution characterization of ovarian cell heterogeneity and intercellular communication. In sheep, large-scale profiling across prenatal (E90), pre-pubertal (M3), post-pubertal (M6), and adult (Y2, Y4) stages identified a conserved ovarian cellular landscape composed of granulosa cells (GCs), oocytes, theca cells, stromal cells, epithelial cells, endothelial cells, immune cells, smooth muscle cells, and mesenchymal cells (Figure 3B) [92]. These data provide a comprehensive cellular framework for understanding ovarian development and folliculogenesis in small ruminants.

In goats, time-resolved single-cell transcriptomic profiling following exogenous human chorionic gonadotropin (hCG)-induced ovulation has revealed rapid changes in follicular cell composition and functional states, including the identification of multiple myeloid subtypes and a transient peak of immune cell infiltration shortly after the luteinizing hormone (LH) surge, highlighting the important role of immune regulation during ovulation [93]. Furthermore, cell–cell communication analyses uncovered extensive interactions among GCs, somatic cells, and immune cells. GCs were found to modulate immune responses through factors such as *IL1RAP*, *COL4A1*, and *COL4A2*, whereas macrophage-derived epiregulin (*EREG*) promoted oocyte maturation, revealing a coordinated signaling network that regulates follicular maturation and ovulation (Figure 3B) [93]. Together, these studies demonstrate that ovarian follicle development and ovulation are highly coordinated processes involving dynamic interactions among germ cells, somatic cells, and immune cells, providing new insights into the cellular and molecular mechanisms underlying female reproductive development in sheep and goats.

### 5.3. Implications for Fertility and Reproductive Efficiency

Beyond their roles in follicular development and ovulation, single-cell transcriptomic analyses have provided important insights into the molecular mechanisms underlying fertility variation and reproductive efficiency in small ruminants. Comparative analyses of animals with different reproductive performances revealed that fertility differences are primarily associated with granulosa-cell (GC) functional states and their interactions with immune cells, particularly macrophages, rather than major alterations in overall ovarian cellular composition [94]. During follicular development, GCs exhibit distinct stage-specific transcriptional programs, with *ASIP* and *ASPN* enriched in early-stage GCs, *INHA*, *INHBA*, *MFGE8*, and *HSD17B1* enriched during follicular growth, and *IGFBP2*, *IGFBP5*, and *CYP11A1* enriched in late-stage GCs (Figure 3B) [95]. These findings suggest that reproductive performance is influenced by the cumulative effects of dynamic regulatory programs throughout folliculogenesis. Consistently, high-fertility Hu sheep exhibited elevated expression of regulatory genes associated with granulosa-cell survival and function, including *FTH1*, *FTL*, and *IGFBP2*, which may promote GC proliferation and steroidogenesis while suppressing follicular atresia through enhanced resistance to ferroptosis and necroptosis, thereby improving oocyte viability and ovulation efficiency [94,96,97]. Furthermore, single-cell transcriptomic profiling of early embryos uncovered key regulatory modules during zygotic genome activation (ZGA), including pathways related to vesicle transport, cytoskeletal dynamics, and cell cycle progression [98].

At the maternal–embryonic interface, bidirectional communication between the conceptus and endometrium is essential for pregnancy establishment. High-resolution single-cell profiling revealed that spherical blastocysts comprise a limited number of cell types dominated by trophectoderm cells, whereas elongated conceptuses undergo extensive cell fate diversification, generating multiple specialized cell populations [99]. This transition is accompanied by enhanced expression of interferon-tau, which plays a central role in maternal recognition of pregnancy. Integrated analyses further identified key ligand–receptor interactions, including IGF2–IGF1R, FGF19–FGFR1, and PROS1–AXL, highlighting complex molecular dialogues between extraembryonic tissues and the endometrium that are essential for successful implantation and early pregnancy maintenance [99].

Collectively, these findings extend single-cell analyses from gametogenesis to early embryogenesis and maternal-fetal interactions, providing an integrated framework for understanding the multistage regulation of reproductive efficiency and offering potential molecular targets for improving fertility in livestock.

## 6. Development of Digestive and Metabolic Tissues at Single-Cell Resolution

The rumen, a digestive organ unique to ruminants, achieves functional maturation through the synchronized development of epithelial cells and the establishment of microbial colonization. Based on large-scale single-cell sequencing and integrated microbiome analysis, researchers have revealed the changes in cellular composition from the embryonic stage to postnatal phases in both sheep and goat rumen, as well as the transcriptional conservation of these processes across species [10]. Furthermore, other researchers, by analyzing rumen samples from Hu sheep during the fetal stages (E30, E60, E90, E110, and E130 days) and postnatal periods (newborn and 45-day-old lambs), identified eight cell subpopulations [9]. These include epithelial cells, fibroblasts, smooth muscle cells, endothelial cells, proliferating cells, lymphocytes, macrophages, and T cells. This study characterized the dynamic development of the rumen epithelium, from its initial non-keratinized state through the formation of regular papillae to full functional maturation. It also proposed that basal cells, keratinocytes, and their differentiated subsets constitute the core cellular foundation for rumen epithelial morphogenesis [9].

Beyond structural development, single-cell transcriptomic analyses have also provided important insights into immune maturation within the developing digestive system. By integrating bulk and single-cell transcriptomic data from the four-chambered stomach (rumen, reticulum, omasum and abomasum) of Hu sheep, researchers demonstrated a progressive activation of innate and adaptive immune responses during early life, indicating that the functional development of the forestomachs and abomasum is initially prioritized toward immune and defense functions [100]. Integrated analyses revealed that T cells, monocytes/macrophages, and endothelial cells are the major cellular contributors to these processes, whereas non-immune cells participate in immune maturation by recruiting immune cells through macrophage migration inhibitory factor (MIF) signaling [100]. These findings provide a comprehensive cellular and molecular framework for understanding early gastrointestinal immune development in ruminants and offer potential targets for nutritional and health-management interventions during the neonatal period.

In addition to stomach, single-cell studies of pancreatic tissue have revealed that the low starch digestibility in ruminants may be associated with relatively low exocrine pancreatic function, particularly deficiencies in amylase expression and the entero-pancreatic regulatory axis. Research indicates that while the exocrine function in adult goats is higher than in newborns, the low expression of the cholecystokinin receptor CCKBR in pancreatic acinar cells and the sparse distribution of CCK-I cells in the duodenum may lead to insufficient signaling for pancreatic enzyme release [101]. This could potentially limit their adaptation to high-energy diets. These findings collectively demonstrate that nutrient digestion and metabolic regulation in ruminants are governed by coordinated, cell-type-specific transcriptional programs across multiple digestive organs. However, comprehensive single-cell characterization of the intestinal tract remains largely lacking in both sheep and goats. In particular, high-resolution cellular atlases of the small intestine and large intestine are still unavailable, limiting our understanding of intestinal cell heterogeneity, nutrient absorption, immune regulation, and host–microbiota interactions in small ruminants.

## 7. Single-Cell Insights into Adipose Tissue Heterogeneity and Adipogenesis

Adipose tissue plays a critical role in livestock production by influencing meat quality, energy metabolism, and environmental adaptation. In sheep, single-cell and multi-omics studies have revealed substantial heterogeneity among adipose depots, including subcutaneous, visceral, and tail fat, each exhibiting distinct cellular compositions and functional characteristics [102]. Depot-specific adipocyte subpopulations have been identified, with visceral and subcutaneous adipose tissues exhibiting distinct adipocyte populations, while tail fat contains specialized fibroblast progenitor cell types [102]. Functional analyses further identified BMP signaling, particularly *BMP5*, as a key regulator of tail fat development and lipid accumulation [102]. Multi-omics integration also revealed that fat deposition is not solely dependent on adipocyte differentiation, but also involves cell–cell communication networks and extracellular matrix remodeling, with candidate regulators such as *SESN1*, *RPRD1A*, and *RASGEF1B* contributing to phenotypic differences in tail fat deposition [103]. Moreover, single-cell analyses of embryonic adipose tissue revealed multiple cellular origins of adipocytes and highlighted enhancer-mediated regulatory mechanisms (e.g., DBI-PPARG) that drive lineage specification and adipogenesis during early development [104]. Collectively, these findings indicate that adipose tissue development is regulated through dynamic interactions among diverse cell populations across developmental stages, providing new insights into the molecular basis of fat deposition in sheep.

In goats, single-cell and single-nucleus transcriptomic studies have provided detailed insights into intramuscular adipogenesis, adipose tissue remodeling, and developmental transitions. In skeletal muscle, distinct subpopulations of fibro-adipogenic progenitors (FAPs) and their differentiation trajectories have been resolved, with ligand–receptor interaction analyses highlighting BMP and IGF signaling as key mediators of intercellular crosstalk, and gene regulatory network analysis identifying *TCF7L2* as a critical transcription factor governing early adipogenic commitment [105]. Beyond muscle, developmental studies revealed dynamic remodeling of adipose tissues. Thermogenic adipocytes undergo progressive whitening, with beige- and white-like adipocytes derived from common progenitors and regulated by signaling pathways such as FGF and CALCR, alongside distinct depot-specific thermogenic capacities [13]. Consistently, postnatal single-cell analyses of perirenal adipose tissue demonstrated a rapid transition from brown adipose tissue to white-like adipose tissue within the first two weeks after birth, driven by shifts in adipocyte subpopulation composition and metabolic pathways, including AMPK and PPAR signaling, reflecting accelerated lipid accumulation and reduced thermogenic capacity [106]. Such developmental transitions may have important implications for livestock production. Increased white adipose deposition may promote lipid storage and intramuscular fat accumulation, thereby improving meat quality traits such as marbling and tenderness [107]. In contrast, the maintenance of brown adipose characteristics may enhance thermogenesis and energy expenditure, which could be advantageous under cold environmental conditions or production systems emphasizing metabolic efficiency [108,109]. These findings suggest that variation in adipose developmental trajectories may influence the balance between carcass quality and energy utilization and could provide potential targets for future precision breeding strategies in goats.

## 8. Single-Cell Studies of Additional Tissues Relevant to Production Traits

### 8.1. Mammary Gland

In mammary gland research, sheep are an ideal model for studying adaptive lactation changes due to their structural similarity to humans. Liu et al. constructed a single-cell atlas of the mammary gland at 60 and 150 days of lactation, identifying multiple epithelial subpopulations, including luminal progenitor cells, hormone-sensing cells, and myoepithelial cells [14]. These cells display distinct differentiation trajectories between peak and late lactation, with epithelial integrity and dynamic extracellular matrix (ECM) remodeling emerging as key drivers of structural and functional changes [14]. Particularly in late lactation, fibroblast-epithelial cell interactions and ECM reconstruction induce significant collagen reorganization, reflecting structural adaptation toward involution and highlighting the role of myoepithelial cells in maintaining functional homeostasis [14]. Cross-species comparisons show high conservation of basal and myoepithelial cells between sheep and humans, while luminal epithelial cells exhibit greater divergence, indicating multi-layered evolutionary adaptations in mammary gland development. Overall, this single-cell atlas provides a cellular-level framework for understanding the regulatory mechanisms of lactation and offers insights into the molecular basis of milk production and mammary health in sheep. However, comparable high-resolution single-cell atlases of the mammary gland are currently lacking in goats, limiting our understanding of the conservation and divergence of mammary developmental programs and lactation-associated regulatory networks across small ruminant species. Future studies generating goat mammary gland single-cell datasets will facilitate cross-species comparisons and advance our understanding of lactation biology in small ruminants.

### 8.2. Horn Buds

Horn buds, as distinctive morphological features unique to ruminants, rely on dynamic coordination among multiple cell types for their formation. In single-cell studies of horn bud development in goats, researchers have for the first time identified the cellular composition and developmental trajectories during the induction (E50), organogenesis (E60), and differentiation (E70) stages, revealing the pivotal role of neural cells in early signal transmission [110]. The formation of neural synapses not only drives the migration of neural crest cells but also collaborates with chondroid cells to participate in keratinocyte differentiation, ultimately promoting the structural formation of the horn bud.

### 8.3. Lung

The lung plays a central role in high-altitude adaptation and respiratory health, serving as the primary organ for oxygen exchange and a key interface for host–environment interactions. The integrative analysis of whole-genome sequencing, bulk RNA-seq, ATAC-seq, and single-cell transcriptomics across multiple tissues revealed dynamic transcriptional responses to hypoxia, identified tissue-specific regulators such as *PARG* and *HMOX1*, and uncovered TAD-constrained cis-regulatory elements that broadly suppress gene transcription under hypoxic stress, while highlighting key adaptive genes including *HIF1A* and *BMPR2*; notably, antenatal hypoxia was shown to enhance hypoxia tolerance in offspring [111]. In parallel, single-cell RNA-seq analysis of sheep lungs following *Mycoplasma ovipneumoniae* infection characterized transcriptomic alterations across 11 cell types, revealing disrupted intercellular communication and immune dysregulation, including impaired CD8^+^ T cell cytotoxicity, depletion of regulatory T cells, and activation of inflammatory pathways such as CypA and MIF signaling; furthermore, the neutrophil marker gene *S100A9* was shown to promote pathogen clearance via ERK pathway activation and ROS production [112]. Collectively, these studies demonstrate the power of integrating multi-omics and single-cell technologies to dissect the molecular mechanisms underlying environmental adaptation and host–pathogen interactions in livestock.

## 9. Limitations and Technical Challenges in Sheep and Goat Single-Cell Studies

### 9.1. Technical and Analytical Challenges in Single-Cell Data

Single-cell omics studies in sheep and goats face several technical and analytical challenges that may affect data quality, biological interpretation, and cross-study comparability. During sample preparation, library construction, and sequencing, technical variability and dropout events frequently occur, leading to false-negative signals when gene expression or chromatin accessibility falls below detection thresholds [57,113,114]. These limitations may be further exacerbated in livestock species because of their high genetic diversity, incomplete genome annotations, and complex transcriptional regulation [59].

A major challenge arises during tissue dissociation. Conventional enzymatic digestion disrupts the native cellular microenvironment and can induce artificial transcriptional responses, resulting in dissociation-associated artifacts [40,41]. Several studies have demonstrated that enzymatic processing may trigger stress-related gene expression programs that do not accurately reflect the in vivo cellular state [115]. Consequently, the process of isolating cells from their native niches may substantially alter transcriptional profiles and influence downstream biological interpretation [40,55]. This issue is particularly relevant in ruminants, where fibrous, lipid-rich, or structurally complex tissues—such as skin, adipose tissue, skeletal muscle, rumen epithelium, and mammary gland—often exhibit low cell recovery, selective loss of fragile cell populations, and increased susceptibility to dissociation-induced artifacts. Therefore, differences among studies may reflect not only biological variation arising from breed, age, developmental stage, diet, or production system, but also technical variation associated with tissue-processing protocols, sequencing platforms, and cell-filtering strategies.

At the analytical level, although more than 1400 computational tools have been developed for scRNA-seq analysis, standardized workflows for non-model livestock species remain limited [116]. Current scRNA-seq technologies typically capture only 10–40% of cellular transcripts, resulting in substantial information loss and increasing the uncertainty of downstream analyses [117]. Moreover, key analytical steps, including quality control, clustering, batch correction, and cell-type annotation, often vary considerably among studies, reducing reproducibility and comparability [118,119]. In sheep and goats, cell-type annotation frequently relies on known marker genes and cross-species projection from human or mouse reference datasets, which may introduce inaccuracies due to evolutionary divergence, incomplete ortholog mapping, and the lack of species-specific reference atlases. Consequently, certain cell populations remain poorly characterized or are classified as “unknown”.

Beyond transcriptomics, integrating multiple single-cell omics modalities presents additional challenges due to differences in data structure, resolution, sparsity, and biological variability across molecular layers. Recent advances in machine-learning and deep-learning frameworks, including contrastive learning–based approaches, have shown considerable potential for integrating heterogeneous datasets and identifying shared cellular states and regulatory relationships across modalities [120]. However, the application of these methods in sheep and goats remains in its infancy and will require the development of comprehensive reference atlases, standardized benchmarking datasets, and species-specific analytical frameworks.

### 9.2. Sample and Trait Coverage Limitations

From the perspective of experimental design and application, current single-cell studies in sheep are often limited by small sample sizes and insufficient representation of breeds and production systems. Most studies focus on a few local breeds or specific developmental stages, lacking large-scale, multi-population comparisons across breeds, environments, and production phases. This restricts the generalizability of findings and limits the identification of robust, conserved regulatory mechanisms. In addition, despite the integration of single-cell atlases for the development of various organs, there is still a lack of systematic single-cell dissection for complex economic traits closely related to production performance, such as muscle growth, fat deposition, feed conversion efficiency, and stress resistance. This limitation somewhat hinders the deeper application of single-cell technology in genetic improvement and molecular breeding of sheep and goats.

### 9.3. Cross-Study Comparability

The most existing studies are predominantly based on scRNA-seq, focusing on cell-type identification, marker gene discovery, and differential expression analysis [121]. However, cellular regulation extends beyond transcriptional profiles to include chromatin accessibility, epigenetic modifications, protein expression, metabolic states, and spatial organization [122]. The limited adoption of multi-omics technologies constrains the ability to uncover the multilayered regulatory mechanisms underlying complex trait formation [75]. Consequently, scientists are continuously exploring new methods for single-cell analysis to provide technical support for revealing the multi-dimensional secrets of cells, including scDNA-seq [123], scATAC-seq [124], single-cell proteomics (e.g., CITE-seq) [125], CRISPR perturbation screens [126], and spatial transcriptomics technologies [127]. The informational content varies across these different modalities, and how to integrate and process such data becomes a key factor influencing research outcomes [128,129].

## 10. Future Prospect and Applications in Precision Breeding

**Figure 4:**
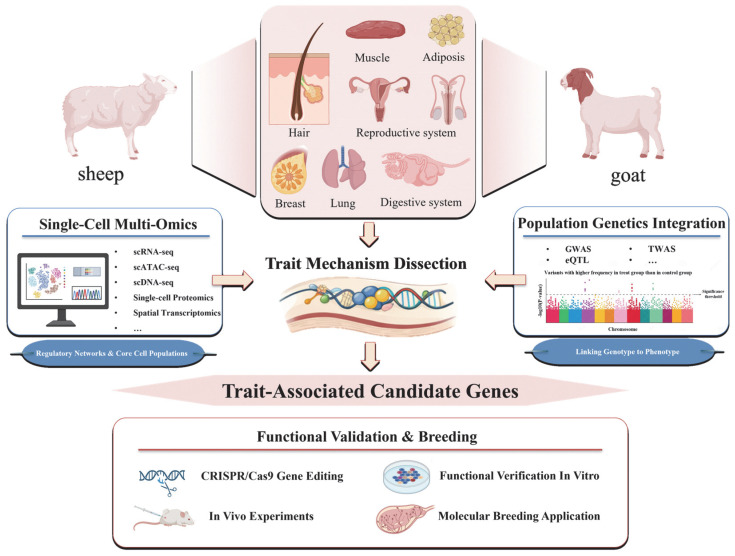
Integrated framework of single-cell multi-omics, genomic selection, and genome editing for precision breeding in sheep and goats. (Materials sourced from bioRender).

Beyond characterizing cellular heterogeneity, single-cell omics studies have identified candidate genes, regulatory pathways, and cell-type-specific mechanisms associated with economically important traits, including wool quality, reproductive performance, feed efficiency, and disease resistance. Future research should prioritize the integration of single-cell transcriptomics with complementary technologies such as scATAC-seq, scDNA-seq, single-cell proteomics, and spatial transcriptomics to simultaneously capture gene expression dynamics, chromatin accessibility, and tissue spatial organization [75,130]. In parallel, integrating single-cell datasets with population genetic approaches, including GWAS [131,132], eQTL [131], and TWAS [133] analyses, will facilitate the identification of cell-type-specific regulatory effects of trait-associated loci and enable more precise mapping of genotype-to-phenotype relationships (Figure 4). Resources such as the Animal QTL Database (Animal QTLdb) provide a valuable framework for linking cell-type-specific expression programs with trait-associated genomic regions [134].

Current evidence demonstrates that several genes associated with economically important traits, including *FGF5*, *BMP15*, *GDF9*, *BMPR1B*, and *MYOT*, have been supported by both single-cell functional studies and genetic association analyses, highlighting their potential roles in regulating wool characteristics, reproductive performance, and meat quality [135,136,137,138,139]. In contrast, relatively limited overlap has been reported for rumen development, feed efficiency, and thermogenic adaptation, highlighting important opportunities for future investigations. Furthermore, the integration of multi-modal datasets across developmental stages will provide a more comprehensive understanding of dynamic cellular interactions and regulatory networks underlying complex trait formation [128,139]. Together, these advances will strengthen our understanding of the molecular basis of economically important traits and accelerate the application of precision breeding, genomic prediction, and genetic improvement strategies in sheep and goats.

Importantly, candidate genes and regulatory networks identified through single-cell analysis can be functionally validated using CRISPR/Cas9-mediated genome editing. Successful applications in sheep, including *MSTN* knockout for enhanced muscle growth [140] and *FGF5* disruption for increased wool length [136], demonstrate the potential of genome editing for livestock improvement. Although single-cell-guided genome editing remains in its infancy in small ruminants, emerging cellular atlases provide unprecedented opportunities to prioritize cell-type-specific targets associated with economically important traits, such as wool production, reproductive performance, adipogenesis, and meat quality. Therefore, integrating single-cell omics with CRISPR/Cas-based functional validation is expected to accelerate the transition from candidate gene discovery to causal inference and precision breeding. Nevertheless, future applications should also carefully consider animal welfare, editing efficiency, off-target effects, regulatory policies, and public acceptance.

## 11. Conclusions

Single-cell omics technologies have substantially advanced the characterization of cellular heterogeneity and tissue development in sheep and goats, particularly in hair follicles, reproductive tissues, rumen epithelium, and selected metabolic organs. More recent studies have expanded these approaches to adipose tissue, skeletal muscle, mammary gland, and immune cells, providing unprecedented insights into the cellular and molecular mechanisms underlying economically important traits. Nevertheless, current applications remain largely restricted to scRNA-seq and are constrained by incomplete genome annotation, challenges in cell-type annotation, limited spatial information, technical biases associated with tissue dissociation, and insufficient representation of breeds, developmental stages, and environmental conditions. Future integration of single-cell transcriptomics with complementary approaches, including single-nucleus RNA sequencing, epigenomics, spatial transcriptomics, and population genetics, will be essential for elucidating cell-type-specific regulatory mechanisms and improving biological interpretation.

The application of single-cell discoveries to breeding is expected to accelerate over the next 5–10 years through the development of livestock cell atlases, improved genome annotations, functional validation of candidate genes, and integration with GWAS and QTL resources. International consortia and standardized data-sharing initiatives will play a critical role in harmonizing experimental protocols, improving sheep and goat reference genome annotations, and enabling cross-breed and cross-species comparative analyses. Such collaborative efforts will facilitate the establishment of comprehensive livestock cell atlases and accelerate the adoption of single-cell technologies in animal science. Importantly, single-cell omics provides opportunities to connect candidate genes and regulatory networks with specific cell types and developmental windows. In wool and cashmere production, these approaches may refine candidate genes associated with follicle density, fiber diameter, wool curvature, and cashmere yield. In reproductive tissues, cell-type-resolved analyses may improve the prioritization of markers related to fecundity, spermatogenesis, and reproductive efficiency. Likewise, studies of adipose tissue, skeletal muscle, and mammary gland can identify regulatory programs associated with intramuscular fat deposition, carcass quality, tenderness, milk production, and lactation performance. These traits have direct economic significance because they influence product quality, feed efficiency, reproductive output, disease resistance, and overall production sustainability.

Overall, single-cell technologies are transforming small-ruminant biology from descriptive tissue-level observations toward cell-type-resolved mechanistic understanding. By integrating single-cell omics with functional genomics, quantitative genetics, and emerging genome-editing technologies, future breeding programs may transition from population-based selection to cellular- and pathway-informed precision breeding strategies, ultimately accelerating genetic improvement and enhancing the sustainability and profitability of sheep and goat production systems.

## Figures and Tables

**Table 1:** Analytical overview of representative single-cell transcriptomic studies in sheep and goats.

Tissue/Trait	Main Biological Objective	Technology/Data Type	Key Biological Findings	Potential Breeding or Husbandry Relevance	Current Limitations/Research Gaps	Representative References
Cashmere goat hair follicle morphogenesis	Resolve cellular heterogeneity and lineage trajectories during follicle development	scRNA-seq	Identified epidermal and dermal lineage programs, dermal papilla heterogeneity, and signaling pathways involved in follicle morphogenesis	Candidate regulators for cashmere yield, wool growth, and fiber quality	Limited spatial validation and limited breed diversity	Ge et al., 2021 [5]
Sheep hair follicle heterogeneity and wool curvature	Characterize cellular composition and molecular features associated with wool curvature	scRNA-seq	Identified heterogeneous skin and follicle cell populations and molecular programs associated with wool structure	Candidate cell types and pathways for wool curvature and fiber-quality improvement	Limited functional validation of candidate genes	Wang et al., 2021 [6]
Dairy goat testis	Construct a cellular atlas of testicular cell types	scRNA-seq	Identified spermatogonia, Sertoli cells, Leydig cells, and other testicular cell populations involved in spermatogenesis	Candidate markers for male fertility and reproductive performance	Limited comparison across breeds and developmental stages	Yu et al., 2021 [7]
Dairy goat male germ-cell and Sertoli-cell development	Characterize developmental patterns of germ cells and Sertoli cells	scRNA-seq	Revealed germ-cell differentiation trajectories and supporting-cell developmental programs during spermatogenesis	Supports identification of fertility-related markers and reproductive management targets	Functional validation remains limited	Ren et al., 2022 [8]
Sheep rumen development	Understand rumen epithelial maturation and digestive adaptation	scRNA-seq	Identified major rumen cell populations and developmental programs associated with keratinization, epithelial differentiation, and digestive adaptation	Potential markers for rumen development, digestive efficiency, and feed utilization	Limited integration with feed-efficiency GWAS/QTL data	Yuan et al., 2022 [9]
Sheep and goat rumen development and host–microbe interaction	Integrate rumen single-cell transcriptomes with microbial/metagenomic information	scRNA-seq and metagenomic integration	Characterized large-scale rumen cellular landscapes and linked host epithelial development with microbial colonization	Supports understanding of fermentation efficiency, rumen health, and nutritional adaptation	Requires functional validation and larger breed/environment comparisons	Deng et al., 2023 [10]
Goat PBMC immune response to parasitic infection	Characterize immune-cell dynamics during infection	scRNA-seq	Resolved dynamic immune-cell responses in peripheral blood mononuclear cells during Haemonchus contortus infection	Candidate immune markers for disease resistance and resilience breeding	Few immune challenge models and limited longitudinal validation	Wang et al., 2024 [11]
Goat PBMC response to Pasteurella multocida infection	Profile systemic immune response after bacterial challenge	scRNA-seq	Identified infection-responsive immune-cell populations and transcriptional changes in goat PBMCs	Potential biomarkers for respiratory disease resistance and animal health	Recently published; requires independent validation	An et al., 2026 [12]
Goat thermogenic adipose tissue whitening	Investigate postnatal transition from thermogenic to white-like adipose tissue	snRNA-seq	Revealed cell-type-specific changes associated with reduced thermogenesis and increased adipogenic/lipid-storage programs	Relevant to energy efficiency, fat deposition, and meat-quality traits	Need direct integration with carcass and production phenotypes	Li et al., 2025 [13]
Sheep mammary gland during lactation	Resolve cellular remodeling and extracellular-matrix dynamics during lactation	scRNA-seq	Identified epithelial, stromal, immune, and extracellular-matrix remodeling programs during lactation	Potential relevance to milk yield, mammary health, and lactation efficiency	Limited datasets across breeds and lactation stages	Liu et al., 2025 [14]

**Table 2:** Early low-throughput single-cell RNA-seq methods.

Method	Key Technical Feature	Advantages	Limitations	Reference
Tang method	Poly(A) tailing and whole-transcriptome amplification	First demonstration of transcriptome profiling at single-cell resolution	Very low throughput; amplification bias	Tang et al., 2009 [15]
STRT-seq	Template switching with 5′-end counting and cell-specific barcodes	Enables multiplexing and transcript counting	Limited transcript coverage; 5′-end bias	Islam et al., 2011 [17]
CEL-Seq	Linear amplification with in vitro transcription and unique molecular identifiers (UMIs)	Reduced amplification bias; good quantitative accuracy	3′-end bias; lower throughput	Hashimshony et al., 2012 [18]
SMART-seq	Template switching for full-length cDNA amplification	Isoform detection and allele-specific analysis	Labor-intensive; lower throughput	Ramsköld et al., 2012 [19]
SMART-seq2	Improved reverse transcription and full-length coverage	High sensitivity and gene detection	Expensive; not suitable for very large studies	Picelli et al., 2013 [23]

**Table 3:** Computational tools and resources for cross-species scRNA-seq annotation in sheep and goats.

Step	Tool/Resource	Purpose	Typical Reference Species	References
Ortholog identification	Ensembl BioMart	Gene conversion and ortholog retrieval	Human, mouse, bovine, pig	Howe et al., 2021 [73]
Ortholog identification	OrthoDB	Comparative genomics and ortholog inference	Multiple species	Kuznetsov et al., 2023 [74]
Data integration	Seurat (FindTransferAnchors)	Anchor-based label transfer	Human, mouse, bovine	Stuart et al., 2019 [75]
Automated annotation	SingleR	Reference-based cell-type assignment	Human, mouse	Aran et al., 2019 [76]
Cell projection	scmap	Projection of query cells onto reference clusters	Human, mouse	Kiselev et al., 2018 [77]
Machine-learning annotation	CellTypist	Automated classification, especially immune cells	Human	Shao et al., 2024 [78]
Batch correction	Harmony	Integration across datasets and species	Multiple species	Korsunsky et al., 2019 [79]
Matrix factorization	LIGER	Identification of shared biological programs	Multiple species	Welch et al., 2019 [80]
Large-scale integration	Scanorama	Integration of heterogeneous datasets	Multiple species	Hie et al., 2019 [81]

## Data Availability

No new data were created or analyzed in this study. Data sharing is not applicable to this article.
